# Surface Length 3D: *Plugin* do OsiriX para cálculo da distância em superfícies

**DOI:** 10.1590/1677-5449.005316

**Published:** 2016

**Authors:** Alexandre Campos Moraes Amato

**Affiliations:** 1 Amato – Instituto de Medicina Avançada, Departamento de Cirurgia Vascular, São Paulo, SP, Brasil.

**Keywords:** validação de programas de computador, *software*

## Abstract

*Softwares* tradicionais de avaliação de imagens médicas, como DICOM, possuem diversas ferramentas para mensuração de distância, área e volume. Nenhuma delas permite medir distâncias entre pontos em superfícies. O menor trajeto entre pontos possibilita o cálculo entre óstios de vasos, como no caso de aneurismas aórticos, e a avaliação dos vasos viscerais para planejamento cirúrgico. O desenvolvimento de um *plugin* para OsiriX para mensuração de distâncias em superfícies mostrou-se factível. A validação da ferramenta ainda se faz necessária.

## INTRODUÇÃO

O *software* OsiriX mostra-se muito útil no planejamento endovascular, com métodos de reconstrução tridimensional multiplanar e projeção de intensidade máxima^1^. Com essas duas técnicas, é possível avaliar comprimento, extensão e diâmetro dos vasos em qualquer ângulo. A reconstrução por volume tridimensional evidencia belas imagens, que têm limitação em uso prático^2^.

**Figura 1:**
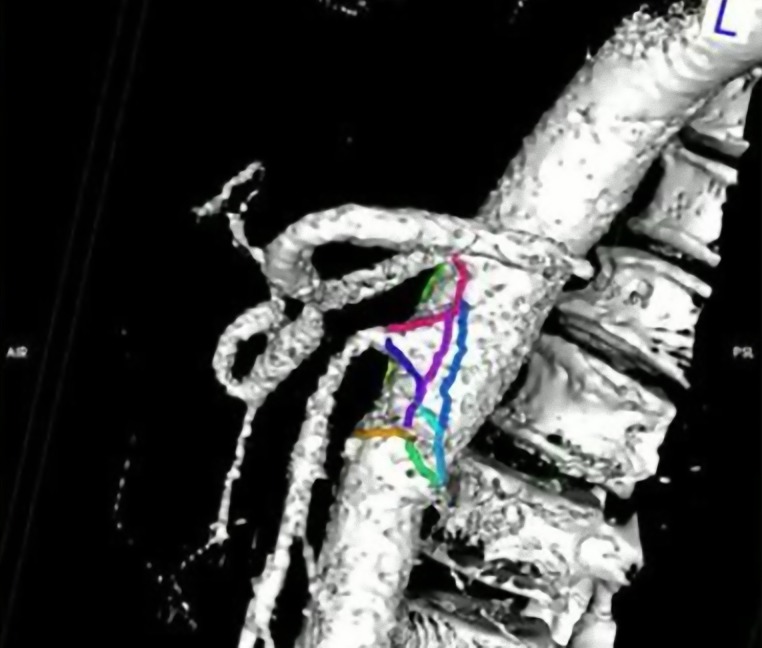
Visualização tridimensional dos tracejados de superfície em modelo de superfície elaborados pelo *plugin* 3D Surface Length 3D e OsiriX.

**Figura 2:**
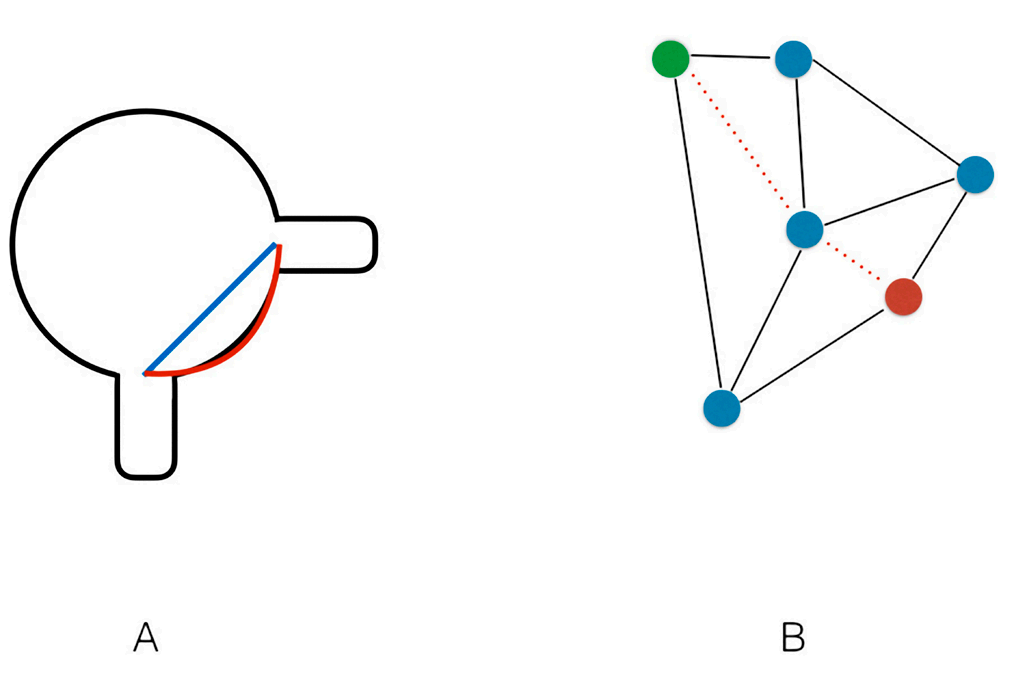
(**A**) Representação bidimensional de corte transversal de vaso mostrando a diferença entre a medida linear e a medida de superfície; (**B**) Representação bidimensional de malha poligonal e algoritmo de cálculo de menor medida entre pontos.

Com o advento da técnica de endopróteses fenestradas e ramificadas e o planejamento cirúrgico aberto de aneurismas de aorta com comprometimento das artérias viscerais, outras informações se fazem necessárias, como o ângulo de saída das artérias e a distância entre elas^3^. A opção entre uma prótese de Coselli e um *patch* para viscerais tem como variável a distância entre essas artérias. A distância linear não pode ser considerada relevante, mas a distância de superfície pode (Figura 1, Figura 2A). Esse foi o problema identificado que conduziu ao desenvolvimento do *plugin*.

## MÉTODO

O *plugin* Surface Length 3D^4^ para OsiriX 3 foi desenvolvido pelo autor em Objective-C para computadores com o sistema operacional OSX com o intuito de calcular distâncias em superfícies e está disponível gratuitamente. O OsiriX é um *software* de visualização de imagens médicas que permite grande liberdade na manipulação das imagens, como também permite a criação de extensões desenvolvidas por terceiros. O método usual de reconstrução tridimensional do OsiriX é a renderização por volume de um conjunto de dados tridimensionais, que consiste em um grupo empilhado de imagens bidimensionais planas. Essas imagens são adquiridas em sequência, com distância padronizada entre si e com um número regular de *pixels* bidimensionais. Os *pixels*, quando tridimensionais, chamam-se *voxels*. Para criar renderização por volume, uma câmera é disposta virtualmente relativa ao espaço criado, e todos os *voxels* passam a conter informação de cor e transparência. A renderização por superfície pode ser feita por diversos algoritmos diferentes e consiste na conversão dos dados tridimensionais em modelos vetoriais, ou seja, modelos com vértices, linhas e planos. Essa conversão depende do algoritmo selecionado, da estrutura a ser convertida e do ponto de corte selecionado. Por causa disso, algumas estruturas são muito bem delineadas na renderização por superfície, como ossos, e outras são muito mal delineadas. Estruturas mal delineadas não possuem consistência na sua densidade ou possuem estruturas adjacentes com densidades semelhantes. O aparelho circulatório não tem as características necessárias para bom delineamento; porém, ao utilizar-se contraste, a densidade se diferencia das estruturas adjacentes, e ele passa a ser devidamente convertido para superfície. Apesar de o método chamar-se renderização por superfície, a segmentação realizada é do contraste vascular; portanto, a parede do vaso pode não estar incluída na superfície.

**Figura 3:**
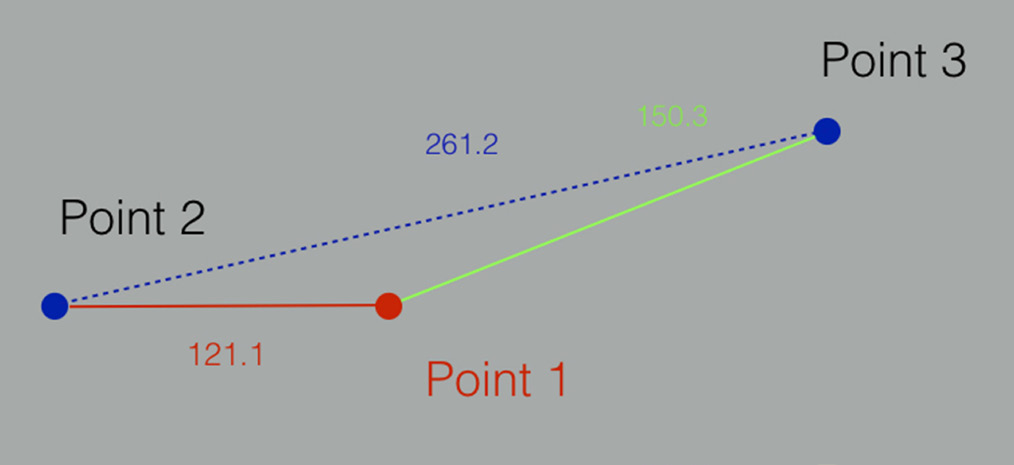
Modelo esquemático apresentado pelo *plugin* dos pontos selecionados e da distância entre eles.

O *plugin* utiliza um algoritmo matemático que busca a menor distância de superfície entre dois pontos e dispõe o resultado planificado esquematizado (Figura 3). O algoritmo utilizado, denominado vtkDijkstraGraphGeodesicPath, calcula a série de linhas que descrevem o menor caminho entre pontos sobre a malha poligonal. O cálculo da distância é feito somando-se as diversas linhas calculadas^5^, baseado no algoritmo de Dijkstra^6^ (Figura 2).

## RESULTADO

O *plugin* criado mostrou capacidade de mensurar distâncias de diferentes estruturas em superfícies devidamente extraídas de *datasets* tridimensionais (Figura 1).

## DISCUSSÃO

**Figura 4:**
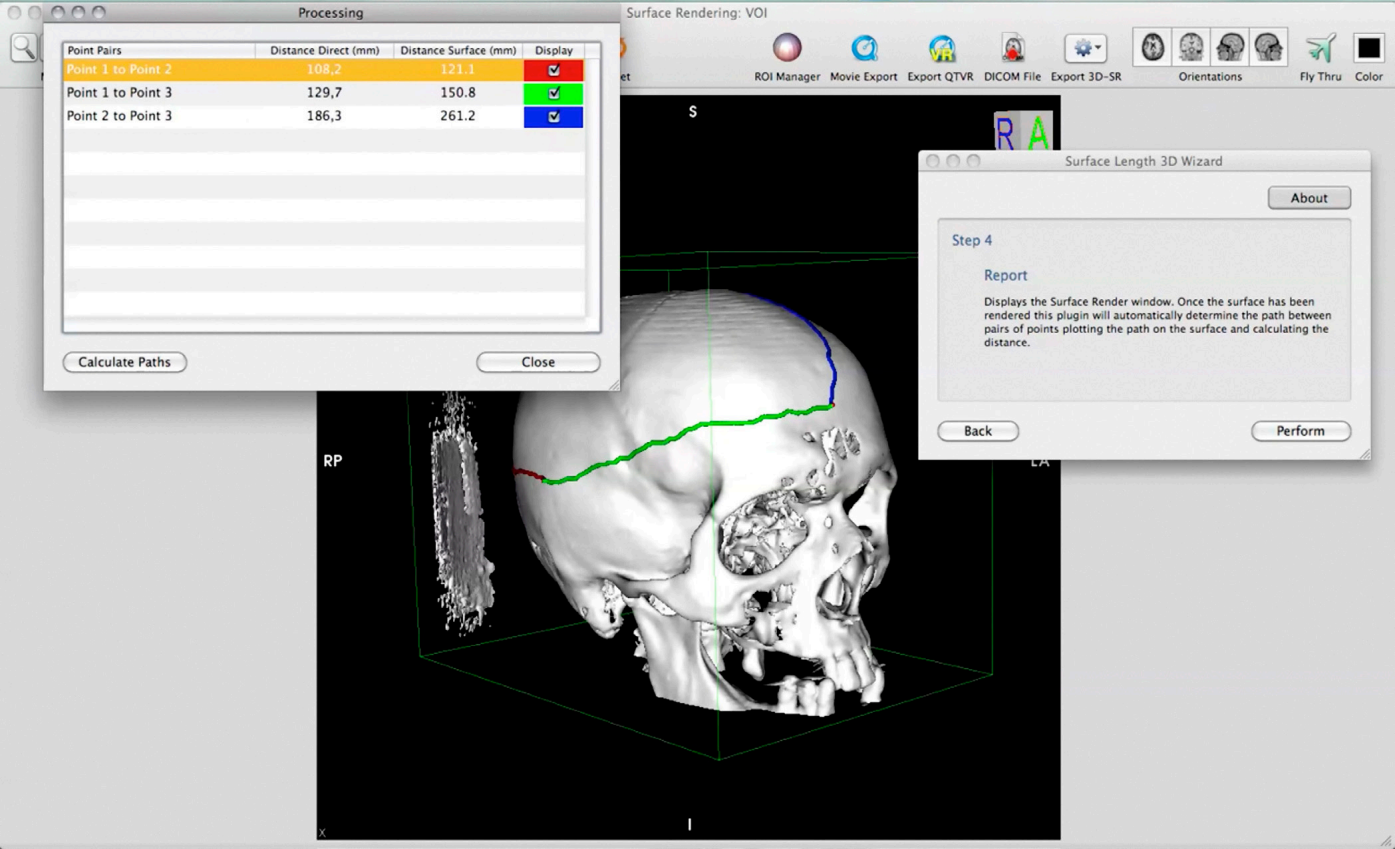
Exemplificação do uso da medida de superfície sobre estrutura óssea craniana.

O objetivo inicial do projeto de fazer medidas de distâncias em superfície mostrou-se factível. A capacidade de identificar aortas e a distância dos vasos viscerais entre si e que necessitam prótese diferenciada tanto para cirurgia tradicional quanto endovascular pode ser uma das possibilidades da ferramenta, mas ainda necessita de avaliação. Durante o período de exposição do método a outras especialidades, notamos possibilidade de uso em neurocirurgia, com a definição de local para drenagem de hematomas subdurais utilizando distâncias mensuráveis de pontos de referência no crânio, fazendo-se a triangulação do local (Figura 4); e na cirurgia plástica, com medidas de superfície entre pontos de referência.

Uma validação prévia das medidas de distância com as ferramentas padrão do OsiriX mostraram precisão de 0,3 mm com boa confiança^7^. Apesar disso, o *plugin* desenvolvido utiliza algoritmo matemático original de cálculo de medida que necessita ser validada para utilização médica. O desenvolvimento da ferramenta e a ampliação de uso para versões atualizadas do OsiriX podem expandir as possibilidades de uso. Novos estudos podem validar o uso da ferramenta com *phantoms* para posterior aplicação prática.

## CONCLUSÃO

A medida de superfície não é técnica conhecida amplamente por não haver outras ferramentas que permitam essa mensuração. Pode ser útil em diversas especialidades médicas, incluindo a cirurgia vascular. Porém, necessita de maiores investigações.
