# Supplementary material for: Surface Length 3D: Plugin do OsiriX para cálculo da distância em superfícies
Source: J Vasc Bras. 2016 Oct-Dec;15(4):308–11. [Article in Portuguese] doi: 10.1590/1677-5449.005316 (PMC5829730; doi:10.1590/1677-5449.005316)
Supplement: Translation

# Translation: Surface Length 3D: An OsiriX plugin for measuring length over surfaces

**Authors:** Alexandre Campos Moraes Amato

**Affiliations:** 1 Amato – Instituto de Medicina Avançada, Departamento de Cirurgia Vascular, São Paulo, SP, Brazil.

**Keywords:** software validation, software

## Abstract

Traditional DICOM medical imaging software offers several tools for measuring distance, area and volume. None allow measurement of distances between points along surfaces. The shortest path between points makes it possible to calculate distances between the ostia of vessels, useful in cases of aortic aneurysms and for assessment of visceral vessels for surgical planning. Developing a plugin for OsiriX for measurement of distances along surfaces proved to be achievable. The tool still needs to be validated.

## INTRODUCTION

OsiriX software has proven very useful for endovascular planning, offering methods for three-dimensional multiplanar reconstruction and maximum intensity projection.^1^ These two techniques enable evaluation of the length, extension, and diameter of vessels at any angle. Reconstruction by three-dimensional volume provides beautiful images, but has limitations for practical use.^2^

The advent of fenestrated and branched stent techniques and planning of open surgery for aortic aneurysms with involvement of visceral arteries demand additional information, such as the exit angles of arteries and the distances between them.^3^ For example, the choice between a Coselli graft and a patch for visceral repairs is based on the variable distance between the arteries. The linear distance is not the relevant dimension, rather it is the surface distance that is needed (Figure 1, Figure 2A). It was identification of this problem that prompted development of the plugin.

**Figure 1:** Three-dimensional view of paths along surfaces on a surface model produced using the Surface Length 3D plugin and OsiriX.

**Figure 2:** (**A**) Two-dimensional cross-sectional representation of a vessel showing the difference between a linear measurement and a measurement along a surface; (**B**) Two-dimensional representation of a polygonal mesh and the algorithmic method for calculating the shortest distance between points.

## METHOD

The Surface Length 3D plugin^4^ for OsiriX 3 was developed by the author in Objective-C for computers running the OSX operating system with the objective of providing a tool for calculating distances along surfaces and is available free of charge. OsiriX is a software package for viewing medical images that offers great freedom for image manipulation and also allows third-party developers to create extensions. The usual method of three-dimensional reconstruction in OsiriX is rendering by volume from a three-dimensional dataset that comprises a piled group of flat two-dimensional images. These images are acquired in sequence, with a standardized distance between each and with a regular number of two-dimensional pixels. Three-dimensional pixels are called voxels. To create volume rendering, a camera is placed virtually relative to the space created and all voxels contain information on color and transparency. Surface rendering can be accomplished using a number of different algorithms and consists of a process by which three-dimensional data are converted into vector models, i.e. models made up of vertices, lines, and planes. The conversion is dependent on the algorithm chosen, on the structure to be converted, and on the cutoff point chosen. As a result, some structures, such as bones, are very well delineated in a surface render, while others are very poorly delineated. Structures that are poorly delineated are those that do not have consistent density or have adjacent structures with similar densities. The circulatory apparatus does not possess the characteristics needed for good delineation, but using contrast its density can be differentiated from the adjacent structures and it can be duly converted in a surface render. Although the method is called surface rendering, segmentation is actually performed on the vascular contrast and so the vessel wall may not be included in the surface.

The plugin employs a mathematical algorithm that seeks the shortest surface distance between two points and displays the result as a flattened schematic (Figure 3). The algorithm used, called vtkDijkstraGraphGeodesicPath, calculates a series of lines that describe the shortest path between points along a polygonal mesh. The total distance is calculated by summing the various different lines calculated,^5^ based on the algorithm developed by Dijkstra (Figure 2).^6^

**Figure 3:** Schematic model produced by the plugin of the points selected and the distance between them.

## RESULTS

The plugin developed proved capable of measuring distances between different structures along surfaces duly extracted from three-dimensional datasets (Figure 1).

## DISCUSSION

The initial objective of the project was to measure distances along surfaces and this proved to be attainable. The capacity to identify aortas and the distances between visceral vessels that need special grafts, whether deployed with traditional surgery or using endovascular techniques, could be one of the tool’s possible applications, but this still needs to be evaluated. During a period in which the method was presented to other specialties, we detected a possibility for applications in neurosurgery, for definition of sites for drainage of subdural hematomas using distances measurable from reference points in the skull to triangulate the site (Figure 4); and in plastic surgery, to conduct surface measurements between reference points.

**Figure 4:** Example application of surface length plugin to measurement of bony structures of the skull.

A preliminary validation of distance measurements using the standard tools available in OsiriX showed a precision of 0.3 mm with good reliability.^7^ Notwithstanding, the plugin developed uses an original mathematical algorithm for calculating measurements that must be validated for medical use. Development of this tool and conversion to make it compatible for use with more up-to-date versions of OsiriX should expand the possible applications. Further studies could be conducted to validate the tool using phantom data before moving on to applications in practice.

## CONCLUSIONS

Measurement along surfaces is a technique that is not widely known because there are no other tools that enable such measurements. It could be useful in many medical specialties, including vascular surgery. However, further investigation is needed.
